# Complete Genome Sequence Analysis of *Bacillus subtilis* T30

**DOI:** 10.1128/genomeA.00395-15

**Published:** 2015-05-07

**Authors:** Shuang-yong Xu, Matthew Boitano, Tyson A. Clark, Tamas Vincze, Alexey Fomenkov, Sanjay Kumar, Priscilla Hiu-Mei Too, Danila Gonchar, Sergey K. Degtyarev, Richard J. Roberts

**Affiliations:** a New England Biolabs, Inc., Ipswich, Massachusetts, USA; b Pacific Biosciences, Menlo Park, California, USA; c SibEnzyme Ltd., Novosibirsk, Russia

## Abstract

The complete genome sequence of *Bacillus subtilis* T30 was determined by SMRT sequencing. The entire genome contains 4,138 predicted genes. The genome carries one intact prophage sequence (37.4 kb) similar to *Bacillus* phage SPBc2 and one incomplete prophage genome of 39.9 kb similar to *Bacillus* phage phi105.

## GENOME ANNOUNCEMENT

*Bacillus subtilis* T30 is the source strain for the methylation-dependent restriction endonuclease (REase) BisI (G^5m^C↓NGC). BisI belongs to the type IIM group of REases that cleave modified DNA (1, 2). BisI strain isolation, its morphological and physiological characterization, as well as the native BisI enzyme property were described previously (3). Here, we report the complete genome sequence of *B. subtilis* T30. Six SMRT cells worth of data from long-insert libraries of *B. subtilis* T30 genomic DNA were obtained. The sequence data were processed using HGAP and Quiver for *de novo* assembly (4). The assembled genome consisted of a single contig of 4.03 Mbp with 4,138 predicted genes (3,896 predicted coding sequences [CDSs]). The *B. subtilis* T30 genome sequence is very similar to that of *B. subtilis* subsp. *spizizenii* W23 (5), except that it contains regions of large repeats that impart difficulty in contig assembly from short reads created by other sequencing methods.

We analyzed the sequence for possible DNA methyltransferases (MTases) and endonucleases in the *B. subtilis* T30 genome by sequence homology analysis with known type I to IV restriction-modification (RM) system components listed in REBASE (1). In addition, by measuring the time-resolved kinetics of dT incorporation opposite to dA or d^m6^A by SMRT sequencing, it is possible to determine the methylation status of the template strand (6). SMRT analysis identified one active type I MTase that must be encoded by the single type I RM system in the genome (*hsdM*, Bis30_13985; *hsdS*, Bis30_13990), as evidenced in the methylated motif 5′ AC^m6^AYN_7_TGNG 3′ (T indicates that the complementary A is modified). The half sites AC^m6^AY and CNC^m6^A are 94.7% and 94.5% modified, respectively, in the sequenced genome for self-protection. By amino acid sequence homology analysis with known DNA MTases, two putative C5 MTases were found in the *B. subtilis* T30 genome. The first, M.BisIII, was active and modified the site CCWGG (Bis30_09930). A second C5 MTase (Bis30_20265) adjacent to the BisI endonuclease was inactive when cloned in *Escherichia coli*. A prophage-encoded HNH endonuclease (Bis30_20225) was found to be active and conferred the DNA nicking specificity of 5′ YG↓GT 3′ in Mg^2+^ buffer (the down arrow indicates the nicking strand as shown). Bis30_20225 nicking specificity is also similar to N.φGamma (5′ CG↓GT 3′) (7, 8). We next evaluated a few open reading frames encoding putative endonucleases. Cell extracts of a putative PLD family endonuclease (Bis30_09935) or purified protein of one HNH endonuclease (Bis30_16040) were inactive in cleaving modified plasmid DNA (pBR322-*fnu4HIM*, G^5m^CNGC, substrate for the native BisI endonuclease) or λ DNA. Thus, Bis30_09935 and Bis30_16040 were excluded as candidates for BisI endonuclease.

### Nucleotide sequence accession number.

The complete genome sequence has been deposited in DDBJ/ENA/GenBank under the accession number CP011051.
